# Metabolic Study of Breast MCF-7 Tumor Spheroids after Gamma Irradiation by ^1^H NMR Spectroscopy and Microimaging

**DOI:** 10.3389/fonc.2016.00105

**Published:** 2016-04-28

**Authors:** Alessandra Palma, Sveva Grande, Anna Maria Luciani, Vladimír Mlynárik, Laura Guidoni, Vincenza Viti, Antonella Rosi

**Affiliations:** ^1^Department of Technology and Health, Istituto Superiore di Sanità, Rome, Italy; ^2^INFN Sezione di Roma, Rome, Italy; ^3^Department of Biomedical Imaging and Image-Guided Therapy, High-Field MR Center, Medical University of Vienna, Vienna, Austria

**Keywords:** MR spectroscopy, spheroids, mobile lipids, radiation, metabolism

## Abstract

Multicellular tumor spheroids are an important model system to investigate the response of tumor cells to radio- and chemotherapy. They share more properties with the original tumor than cells cultured as 2D monolayers do, which helps distinguish the intrinsic properties of monolayer cells from those induced during cell aggregation in 3D spheroids. The paper investigates some metabolic aspects of small tumor spheroids of breast cancer and their originating MCF-7 cells, grown as monolayer, by means of high–resolution (HR) ^1^H NMR spectroscopy and MR microimaging before and after gamma irradiation. The spectra of spheroids were characterized by higher intensity of mobile lipids, mostly neutral lipids, and glutamine (Gln) signals with respect to their monolayer cells counterpart, mainly owing to the lower oxygen supply in spheroids. Morphological changes of small spheroids after gamma-ray irradiation, such as loss of their regular shape, were observed by MR microimaging. Lipid signal intensity increased after irradiation, as evidenced in both MR localized spectra of the single spheroid and in HR NMR spectra of spheroid suspensions. Furthermore, the intense Gln signal from spectra of irradiated spheroids remained unchanged, while the low Gln signal observed in monolayer cells increased after irradiation. Similar results were observed in cells grown in hypoxic conditions. The different behavior of Gln in 2D monolayers and in 3D spheroids supports the hypothesis that a lower oxygen supply induces both an upregulation of Gln synthetase and a downregulation of glutaminases with the consequent increase in Gln content, as already observed under hypoxic conditions. The data herein indicate that ^1^H NMR spectroscopy can be a useful tool for monitoring cell response to different constraints. The use of spheroid suspensions seems to be a feasible alternative to localized spectroscopy since similar effects were found after radiation treatment.

## Introduction

Solid tumors grow in a three-dimensional (3D) spatial conformation, resulting in an heterogeneous exposure to oxygen and nutrients, which induces radio- and chemotherapy resistance with a high rate of tumor recurrence and treatment failure. Different model systems have been proposed in order to gain insight into the mechanisms that underlie induced tumor resistance (1). To investigate the characteristics of tumors and their response to chemo–radio treatment, cells obtained from tumors are usually cultured as a monolayer, though differences with the original tumors have been observed (2). Three-dimensional *in vitro* models, such as spheroids, have been used in cancer research as an intermediate model between *in vitro* cancer cell line cultures and *in vivo* tumors. Spherical cancer models are major 3D *in vitro* models that have been mostly described over the past four decades (1). Spheroids provide an *in vitro* 3D model for studying proliferation, cell death, differentiation, and metabolism of cells in tumors and their response to different treatments (3). Spheroids above 500 μm in diameter commonly display a layer-like structure comprising a necrotic core surrounded by a viable rim, which consists of an inner layer of quiescent cells and an outer layer of proliferating cells (4). Cellular microenvironments, such as hypoxia, which has been identified as a cause of drug resistance can be modeled and created within spheroids for accurate testing of drug efficacy. In fact, the cells in the spheroid core may mimic the condition of hypoxia typical of solid tumors (5–7). Spheroids have often been proposed as a model in view of their potential use in preclinical studies (8), for example, for studying tumor stem cells, such as those growing as neurospheres from brain tumors (9) or as mammospheres from breast cancer (10). Obviously, in these systems, it is critical to distinguish between cell aggregation-related phenomena from phenomena associated to the stem nature of the cells. Spheroids have also been studied to better understand the energy metabolism of breast cancer cells, where an unexpected modulation of glycolysis and mitochondrial metabolism has been observed and used as targeted anti-mitochondrial therapy (11).

Magnetic resonance spectroscopy (MRS) and imaging (MRI) are versatile techniques to study tissues and related models, such as multicellular 3D systems (12). They may represent a useful tool to elucidate cell metabolism for they can reveal biomarker metabolites to be targeted for disease treatment without perturbing the system (11, 13). Both approaches are valid. Through high-resolution (HR) MRS, it is possible to monitor cell metabolism in systems much more complex than a single cell, whereas MRI and localized MRS allow the separate monitoring of the metabolism in the inner region and in the peripheral cell layers where DNA synthesis and mitotic activity are largely confined.

In a previous paper, ^1^H NMR was used to reveal differences in signals of lipids and lipid metabolites during cell growth in culture. High intensity mobile lipid (ML) signals were found during the first days in culture; afterwards the same signals declined but started to increase again at late confluence in MCF-7. Spectra from suspensions of MCF-7 spheroids suggested that lipid metabolism in spheroids mimics that of confluent cultures of monolayer cells (2). In the present work, we studied small MCF-7 spheroids in suspension by HR ^1^H NMR, and single larger MCF-7 spheroids by localized ^1^H MR spectroscopy. Spectra were compared with those of MCF-7 cells growing as monolayer under normal and hypoxic conditions. In addition, given the well-known higher resistance of hypoxic tumor cells to radiation therapy, we investigated spheroid response to irradiation with a single acute 20 Gy dose of gamma rays to get information on the possible effects of radiation therapy on tumors. We irradiated cells and spheroids with a single high radiation dose comparable with total doses delivered during radiation therapy in a fractionated regimen, and with single high doses used in other therapeutic modalities, such as intraoperative radiotherapy and stereotactic radiosurgery (14). In particular, NMR visible lipids and glutamine (Gln)–glutathione (GSH) metabolic pathways were examined in consideration of their role in the cell response to oxidative stress.

## Materials and Methods

### Cells and Spheroids

MCF-7 cells were purchased from ATCC (Manassas, VA, USA) and kindly donated by Dr Stefania Meschini, Istituto Superiore di Sanità, Rome (Italy). Both monolayer cultures and spheroids were grown in RPMI 1640 medium (Hyclone, Logan, UT, USA) supplemented with 10% fetal calf serum (Hyclone, Logan, UT, USA) and 50 μg/ml gentamicin. Monolayer cells were routinely seeded in 175 cm^2^ flasks in 50 ml medium. The medium was replaced every 72 h.

Spheroids were obtained by growing cells in a gyratory rotation system at a rate of 70 rpm, at 37°C: a suspension of MCF-7 cells was inserted in a 225-ml conical tube filled with 180 ml growth medium, at a density of 4 × 10^6^ cells per tube and maintained during spheroids aggregation in a horizontal position.

Cells and spheroids were irradiated in culture flasks at a dose of 20 Gy with a ^137^Cs gamma rays source [Gammacell 40 Exactor (NORDION, Canada)]. Cells were harvested and measured at 48 h after irradiation.

Hypoxia was obtained by growing cells for 24 h in 2% O_2_, 93% N_2_, and 5% CO_2_.

For HR spectroscopy experiments, NMR cell samples were prepared as described elsewhere (15). The pellet from monolayer cells, suspended in phosphate-buffered saline (PBS) (approximately 20 × 10^6^ cells), was transferred into a coaxial tube system. Spheroid suspensions were centrifuged at 400 rpm for 3 min and washed in PBS, the pellet was then transferred into the coaxial tube system. In both systems, 1 mM sodium 3-(trimethyl-silyl) propionate 2,2,3,3-d (TMSP) in D_2_O in the external compartment was used to provide a lock signal and a frequency standard.

For microimaging and localized spectroscopy experiments, individual spheroids were selected from the spinner tube, suspended in PBS, and poured into a 1% (w/v) low-melting-point agarose solution. Agarose powder (Sigma chemicals, Type VII, low gelling temperature) was dissolved in deionized water and heated in a microwave oven, until all agarose was completely dissolved. The solution was allowed to cool to 37°C, then the spheroid suspension was added. Small amounts of the solution, containing one or more spheroids, were inserted in capillary tubes (2 mm diameter) and allowed to solidify at room temperature.

### ^1^H NMR Spectroscopy High-Resolution ^1^H MRS Measurements

^1^H MR spectra were run at 400.14 MHz on a digital Avance spectrometer (Bruker, AG, Darmstadt, Germany). One-dimensional ^1^H NMR spectra were acquired with a 90° RF pulse, using a sweep width of 4006.4 Hz. Typically 300 scans were sufficient to obtain a good signal-to-noise ratio for intact cells.

Two-dimensional ^1^H correlation spectroscopy (COSY) spectra of cells were acquired using a 90° − *t*_1_ to 90° − *t*_2_ pulse sequence, by summing 16 free induction decays for each of 256 increments in *t*_1_.

Spectra were acquired as a matrix of 512 × 256 data points in the time domain. A Lorentzian–Gaussian function with LB1/4:10 Hz and GB1/4: 0.1 was applied to enhance the resolution in the *t*_1_ domain before Fourier transformation. Water suppression in one- and two-dimensional ^1^H experiments was achieved by irradiating water signal. Measurement of cell samples lasted approximately 100 min (10 min for one dimensional and 90 min for the two-dimensional experiment) as previously reported (2). Cell viability, tested by trypan blue exclusion, was greater than 90% at the beginning of the preparation and greater than 80% at the end of the MRS measurements, in agreement with the literature for other cell samples examined with MRS under similar conditions (15). One-dimensional spectra were run before and after two-dimensional COSY to check the stability of the signals of interest. Cell spectra were run on samples containing comparable cell mass.

### Quantitative Analysis of 1D Spectra and 2D COSY Cross Peak Integration

Resonance deconvolution of one-dimensional spectra was performed using Bruker software “1D WINNMR” (Bruker, Germany). A Gauss/Lorentz ratio equal to unity for the line shape function was chosen. Nine deconvolution lines were used to obtain a good fit in the interval 0.75–1.65 ppm as previously reported (9). All peak positions were fixed, while peak intensities and line widths were fitted. No prior knowledge was imposed on –(CH_2_)_n_– of ML resonance. The quantitative analysis of peaks was done by measuring the fitted peak intensities. Individual integral values were normalized using the methyl group of cytosolic polypeptides at 0.94 ppm as internal reference. This signal, which is observed in cancer cells of different origin as well as in normal tissues, belongs to the methyl group of cytosolic polypeptides, and is indicative of cell mass (16).

Two-dimensional cross-peak integration was done by the Bruker “2D WINNMR” software (Bruker, Germany). The integral values were computed by summing all positive points inside the integral region. The plane baseline was evaluated and subtracted from the integrals. Besides providing spectral assignment from correlations between pairs of related resonances that overcame the problem of peak superimposition in 1D spectra, the off-diagonal elements in 2D spectra gave information on relative concentrations. Cross-peak integrals were normalized to the intensity of lysine (Lys) cross peak at 1.70–3.00 ppm. This peak is reasonably constant in a large number of cell models and tissue samples (9, 17).

### Localized ^1^H MR Spectroscopy and Imaging

^1^H MR localized spectroscopy and imaging were done with a Bruker AVANCE spectrometer microimaging device, equipped with a 2 mm inner diameter resonator. Proton density weighted images were acquired using a multislice spin-echo (MSME) pulse sequence with the following parameters: 0.5 cm field of view, 0.3 mm slice thickness, 2000 ms repetition time, 15 ms echo time, and 256 × 256 acquisition matrix. Localized spectra were acquired from a volume of interest of 0.3 mm × 0.3 mm × 0.3 mm using the PRESS sequence with 15 ms echo time and 3600 acquisitions (100 min total acquisition time). Exponential line broadening was used resulting in a line width of 30 Hz.

## Results

**Figure 1:**
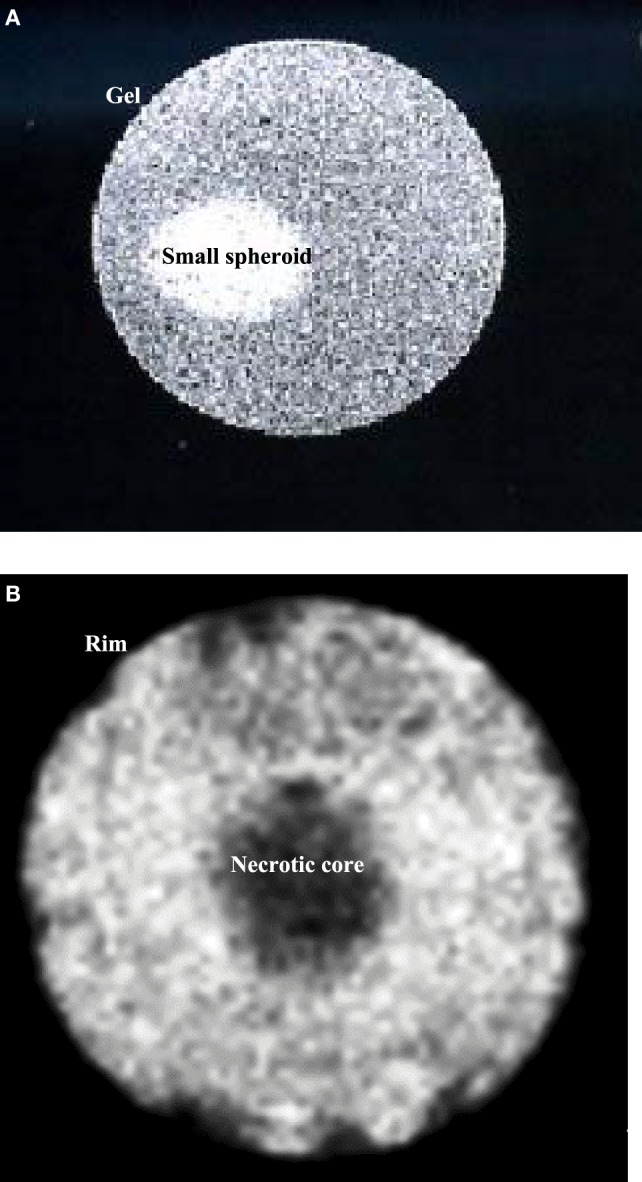
**(A)** Sagittal image of a small spheroid with a diameter of about 0.5 mm, inserted in agarose gel. **(B)** Sagittal image of a spheroid after 10 days in culture. The diameter is about 1.8 mm. It is possible to identify two different regions: a necrotic core and a viable rim. Both images were acquired with an MSME sequence.

Magnetic resonance images of MCF-7 spheroids of different sizes were obtained and compared. Figure 1A depicts a representative small spheroid, <0.5 mm diameter, usually obtained after 4 days in culture. Figure 1B shows a larger spheroid, 1.8 mm diameter, typically obtained after at least 10 days in culture. In this latter case, a necrotic core is clearly visible, while cells in the rim look similar to those of small spheroids.

To avoid the confounding presence of the necrotic core characterized by non-viable cells with a metabolic fingerprint substantially different from the cells in the rim, we examined only small spheroids by both localized and HR MR spectroscopy. When using an internal signal as a reference for quantification, the number of cells *per se* does not influence metabolite levels. On the other hand, different spheroid sizes, due to different number of cells, may influence spectral signals because of differences in pO2 gradient within spheroids. For this reason, a lot of attention was paid to keep the spheroid size constant. The size conformity was achieved by the strict control of growth conditions and was confirmed by light microscopy and MR imaging.

**Figure 2:**
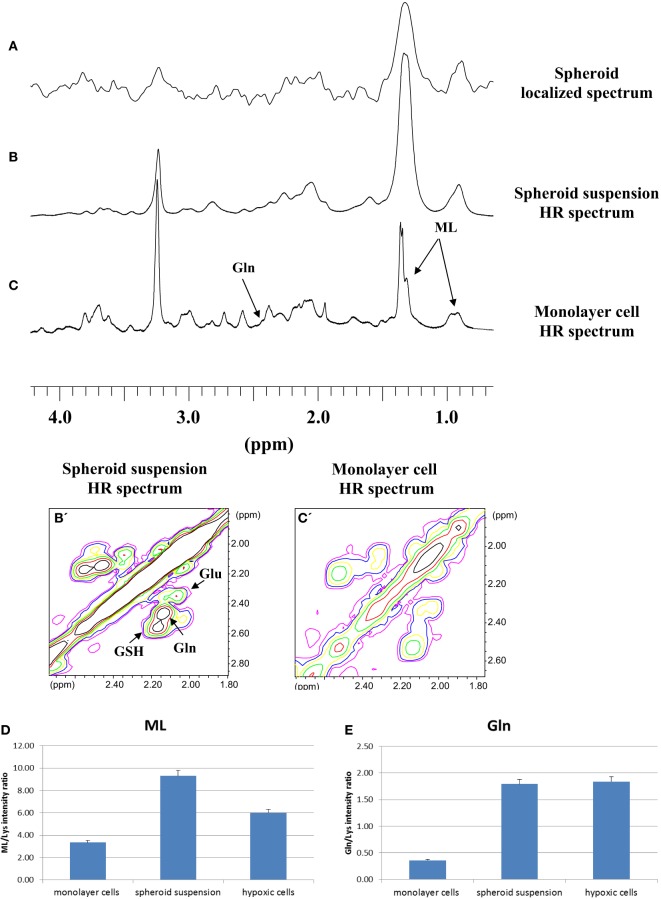
**(A)** Localized 1D spectrum of a single small MCF-7 spheroid. 1D and 2D COSY high resolution (HR) spectra of small MCF-7 spheroid suspension **(B,B’)** and of monolayer MCF-7 cells **(C,C’)**. Cross-peak labeled ML is from terminal methyl and bulk methylene coupling in mobile lipids (ML). Signal intensities of ML **(D)** and Gln **(E)** from 2D spectra of monolayer MCF-7 cells, spheroid suspension, and MCF-7 cells grown under mild hypoxia conditions (average of five experiments, error is the SD). In the figure, GSH and Glu stand for glutathione and glutamate, respectively. TMSP in D_2_O in the external compartment was used to provide a lock signal and a frequency standard.

Localized spectra of single small spheroids were then obtained. A representative spectrum is shown in Figure 2A. These spectra were compared with HR NMR spectra from a suspension of small spheroids and cells grown as monolayer. Figures 2B,B’,C,C’ show typical HR 1D and 2D COSY spectra of small spheroid suspensions and monolayer MCF-7 cells, respectively. Interestingly, the localized spectrum of the single spheroid (Figure 2A) showed remarkable similarities with the HR spectrum of the small spheroid suspension (Figure 2B); in both cases signals at 1.28 and 0.89 ppm dominated the spectra. These signals are attributed to MLs, mostly triglycerides in intracellular lipid droplets (18, 19). The greater signal linewidth in spheroid (Figures 2A,B) vs. monolayer cell spectra (Figure 2C) can be explained by a lower tumbling of lipids aggregates in spheroids due to their immobilization in the three-dimensional structure. Gln signal at 2.44 ppm was also quite different in spheroid and monolayer cell spectra.

Given the similarities between the localized and small spheroid suspension spectra, we studied the suspension by HR spectroscopy, preferentially using 2D COSY, that was recently implemented even for *in vivo* spectroscopy (19), for more precise quantification of spectral changes. In the present paper, MLs were quantified by cross-peak integrals in 2D COSY spectra deriving from the interaction of the terminal methyl group at 0.89 ppm and the proximal methylene at 1.28 ppm, excluding the contribution from ω-3 fatty acids, where methyl protons at 0.98 ppm are coupled to the allylic methylene at 2.09 ppm (18, 19). The results of the quantification of ML and Gln signals in 2D spectra are shown in Figures 2D,E. Assignments of the signals of interest were performed on the basis of comparison with 1D and 2D COSY spectra of total lipid extracts and of Gln pure compound, reported in Supplementary Material (Figure S1 and S2 in Supplementary Material, respectively).

The spectra of spheroid samples were characterized by higher intensity of ML (Figure 2D), mostly neutral lipids, and Gln signals (Figure 2E) as compared with their monolayer cells counterpart, most likely due to the occurrence of lower cell oxygenation in spheroids. To assess whether the behavior of these signals could be attributed to lower cell oxygenation in spheroids, we measured the spectra of MCF-7 monolayer cells grown under mild hypoxic conditions (Figures 2D,E). In both spheroids and hypoxic cells spectra, higher concentrations of Gln were found with respect to the spectra of monolayer cells (Figure 2E). A similar effect was observed in hypoxic HeLa cells (20). Also, the ML signals were more intense in comparison with well oxygenated cells growing as monolayer (Figure 2D).

**Figure 3:**
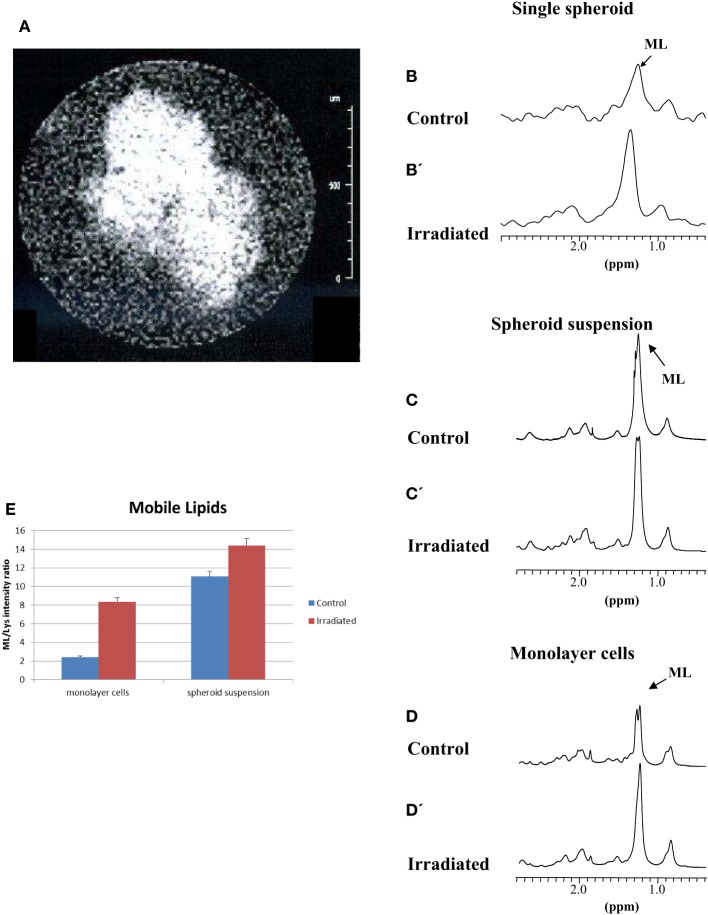
**(A)** MR image of a typical irradiated spheroid 48 h after a 20 Gy dose irradiation. **(B,C,D)** Spectra of control and, **(B’,C’,D’)**, spectra of an irradiated single spheroid, a spheroid suspension and monolayer cells 48 h after a 20 Gy dose irradiation. **(E)** Quantitative evaluation of ML signal intensities in 2D COSY spectra of monolayer cells and the spheroid suspension (average of five experiments, error is the SD). The most evident metabolites are labeled and abbreviated as in Figure 2.

We then examined the effects of irradiation in this multicellular model for possible differences compared to monolayer cells. Spheroids suspension has been irradiated with a dose of 20 Gy. Under these conditions, cell proliferation is completely arrested, although maintaining metabolic activity. Monolayer MCF-7 cells were examined in the past at different times after irradiation (18). In the present paper, we examined samples 48 h after irradiation. Based on our previous experience (18), at this time interval, lipid signals in irradiated samples are much more intense than those in control samples, the latter still being in exponential growth. At later time intervals, exponential growth cannot be assured for control samples and consequently differences with irradiated samples can be deceptive. No cell killing was observed within 48 h after irradiation (21). Therefore, changes in lipid and Glx pool metabolism could be observed. Figure 3A shows the MR image of a typical spheroid 48 h after irradiation. Irradiated spheroids lose their regular shape, appearing larger and frayed. The effect of irradiation on ML signals is shown in Figures 3B,B’,C,C’,D,D’ for a localized single spheroid, spheroid suspension, and monolayer cell spectra, respectively. The effect on single spheroid and on spheroid suspension was similar to that on monolayer cell spectra. The irradiation produced a strong increase in ML signals in all cases (Figures 3B’,C’,D’). Figure 3E reports relative intensities of the ML signal in 2D COSY spectra of monolayer cells and spheroid suspensions, which shows the increase of ML in irradiated samples (average of five experiments).

**Figure 4:**
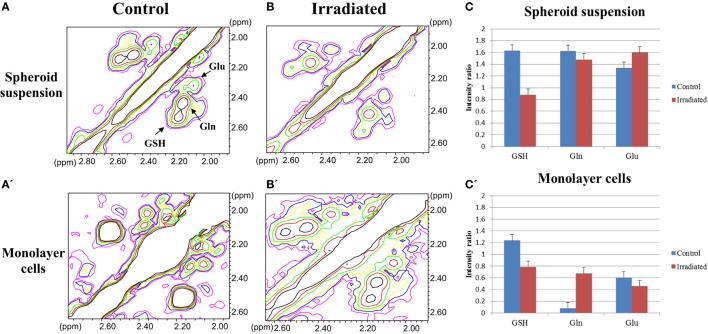
**(A,A’,B,B’)** The Glx pool region of 2D COSY spectra of control and irradiated spheroid suspensions and monolayer cells after 48 h of 20 Gy dose irradiation. **(C,C’)** Quantitative evaluation of GSH, Gln, and Glu signal intensities in 2D COSY spectra of spheroid suspension and monolayer cells (average of five experiments, error is the SD), respectively. The most evident metabolites are labeled and abbreviated as in Figure 2.

In addition, taking into account the oxidative stress induced by irradiation and the antioxidant properties of GSH, we analyzed the Glx pool (GSH, Gln, and Glu) region in 2D COSY spectra from spheroid suspension and monolayer cells. Figure 4 shows the effect of irradiation on signals of the Glx pool; a representative experiment is reported in Figures 4A,A’,B,B’ and the quantification of five experiments in Figures 4C,C’ for spheroid suspension and monolayer cells, respectively. GSH decreased in both spheroids and monolayer cells upon irradiation, the effect being very strong in spheroids, while Gln remained high in irradiated spheroids and increases in monolayer cells after irradiation.

## Discussion

According to several studies and recent reviews (1, 22), some important properties of cell biology are shared by multicellular cancer spheroids and solid tumors *in vivo*, in particular growth characteristics, including growth rates (23), microenvironmental conditions, such as hypoxia, and related resistance to radiation treatments (1).

^1^H NMR spectroscopy has provided new insights into cell metabolism in animals and humans as it allows investigation of biologically active molecules in cells. With this respect, cell aggregates, such as spheroids, provide a system of higher complexity as compared with monolayer cells allowing better characterization of cell metabolism. In the present work, we compared the localized spectrum from a voxel in a single spheroid with NMR spectra from spheroid suspensions. A similar spectral behavior indicates that spheroid suspensions can be useful in NMR-based metabolic studies, thanks to simple sample preparation and spectra measurement.

The intense lipid signals attributed to fatty acid chains in neutral lipids (ML) have elicited much attention in the NMR community, which aims at providing new tools to improve the knowledge of lipid metabolism and correlate the spectral observations to biologically relevant events (24). Several studies have been conducted on these signals. Some authors found ML increasing concurrently with cell death by apoptosis (25). By contrast, such correlation was not found in our previous studies (18, 26). In fact, the intense increase in lipid signals observed in the monolayer MCF-7 cells after irradiation did not correlate with the onset of consistent apoptosis. Furthermore, HeLa cells, which had undergone relevant apoptosis after irradiation, did not show any increase in ML signal (18, 26). No definitive statement on the origin of ML accumulation or degradation may be issued given the complex, interrelated reactions involving lipid synthesis and degradation in different cells, and the numerous changes taking place in response to stress of different origin. On the other hand, ML signals were found to be sensitive in detecting cellular responses to microenvironmental changes, such as irradiation or treatment with anticancer drugs. Thus, they are good candidates for detecting success or resistance to treatments (27).

Lipid signals (ML) were found to be more intense in multicellular spheroids than in monolayer cells: this can be attributed to lower cell oxygenation with the ensuing decline in cell growth as that observable in solid tissues (1). A similar increase in ML signals was confirmed by growing monolayer cells under low oxygen conditions, since a relevant increase in the intense lipid signal is present in hypoxic cells, possibly related to impaired fatty acid beta-oxidation due to the shift from aerobic to anaerobic metabolism (28). Similar observations were obtained in hypoxic HeLa cells (20). In addition, the hypoxic condition has been associated with triglyceride and free fatty acid accumulation (29). Hypoxia is expected to increase lactate production, therefore, decreasing environmental pH: culturing in acidic pH produced an increase in lipid signals in C6 glioma cells (30). The ML increase may then be also related to decreased pH.

Finally, we observed an increased ML content in the cells after the radiation treatment. Interestingly, this has been reported for cells exposed at high doses, such as those used in medical treatments, not a general phenomenon though, as it is modulated by differences in radiation sensitivity (18, 31). In a previous work on MCF-7 cells grown as monolayer cultures, we had already observed more intense ML signals in the spectra of irradiated cells with respect to control samples (18). This was attributed to cell cycle-dependent impairment of phospholipid synthesis and the consequent triglyceride accumulation accompanying the radiation-induced cell cycle block and proliferative arrest. Both in monolayer cell and in spheroid suspensions, lipid signals increased after irradiation. The effect in monolayer cells was attributed mainly to a slowing down of MCF-7 cell growth since apoptosis was not significant in these irradiated cells (21). Irradiated spheroids showed intense ML signals (likely related to the enhanced storage of lipids) and loss of their regular shape; while in monolayer cells, an altered cell growth condition was observed in the past (18).

Exposure to radiation usually affects metabolites of the Glx pool involved in antioxidant reactions of the cell response to oxidative stress. The analysis of Glx pool metabolites showed a decrease in GSH signal intensity after irradiation in both monolayer cell and spheroid spectra. The decrease of GSH in spheroids after irradiation is due to its use as an antioxidant against reactive oxygen species (ROS) produced in large quantity by irradiation. The role of GSH in the radiation response of mammalian cells is well known and a protective effect was actually demonstrated in irradiated MCF-7 cells grown as monolayers (21). Similar effects were observed in hypoxic cells (32). Gln is differently regulated in monolayers and in 3D structures, being more abundant in spheroids than in cells growing as monolayer. This effect may also be ascribed to the reduced oxygen availability in spheroids where the lower oxygen tension induces increased Gln levels, as much as it occurs in other cell aggregates, such as stem cell neurospheres (9). This behavior can be attributed to upregulation of Gln synthetase and downregulation of glutaminases found in hypoxic conditions (33).

Although it has long been known that cell radiosensitization *in vitro* is easily done by depletion of antioxidant scavengers, such as GSH (21, 34, 35) and that this molecule may be involved in antioxidant defense in different cultured cells (25), it is still under discussion whether targeting cancer cells by ROS-mediated mechanisms may be a reasonable therapeutic approach (36, 37). Modulation of antioxidant defenses of cancer cells by radio/chemo therapies may, in principle, provide a tool to improve therapeutic effectiveness. In this context, it would be worth further exploring the role of GSH together with that of amino acids Glu and Gln involved in its synthesis during therapeutic treatments.

## Conclusion

The comparison between localized ^1^H MR spectroscopy of single spheroids and HR NMR on spheroid suspensions suggested the use of the latter for studying cell metabolism in spheroids, used as a 3D model close to solid tumors. Tumor tissues are often characterized by intense signals from fatty acids that have also been found in spheroids and, with less intensity, in the spectra of monolayer cells. These signals are also intense in irradiated and hypoxic MCF-7 cells, which is likely related to switching toward glycolytic metabolism after the radiation treatment. Gln levels are also quite high in spheroids as compared to 2D cultures. Similar Gln levels were present in different 3D aggregates, such as neurospheres. The Glx pool is strictly involved in the cell response to the hypoxic condition and radiation treatment, showing a GSH decrease due to its antioxidant role in irradiated samples of either spheroids or monolayer cells.

## Author Contributions

AP designed the work, and carried out NMR experiments and data analysis. SG carried out NMR experiments and data analysis. AL and AR carried out cell cultures and spheroid growth. VM carried out MR microimaging and localized spectroscopy experiments. LG, VV, and AR contributed to the interpretation of data for the work. All authors discussed the results, and read and approved the final manuscript.

## Conflict of Interest Statement

The authors declare that the research was conducted in the absence of any commercial or financial relationships that could be construed as a potential conflict of interest. The reviewer SB and handling Editor declared their shared affiliation, and the handling Editor states that the process nevertheless met the standards of a fair and objective review.
