# Supplementary material for: Metabolic Study of Breast MCF-7 Tumor Spheroids after Gamma Irradiation by 1H NMR Spectroscopy and Microimaging
Source: Front Oncol. 2016 Apr 28;6:105. doi: 10.3389/fonc.2016.00105 (PMC4848320; doi:10.3389/fonc.2016.00105)
Supplement: Supplementary file 1 [file Image_1.PDF]

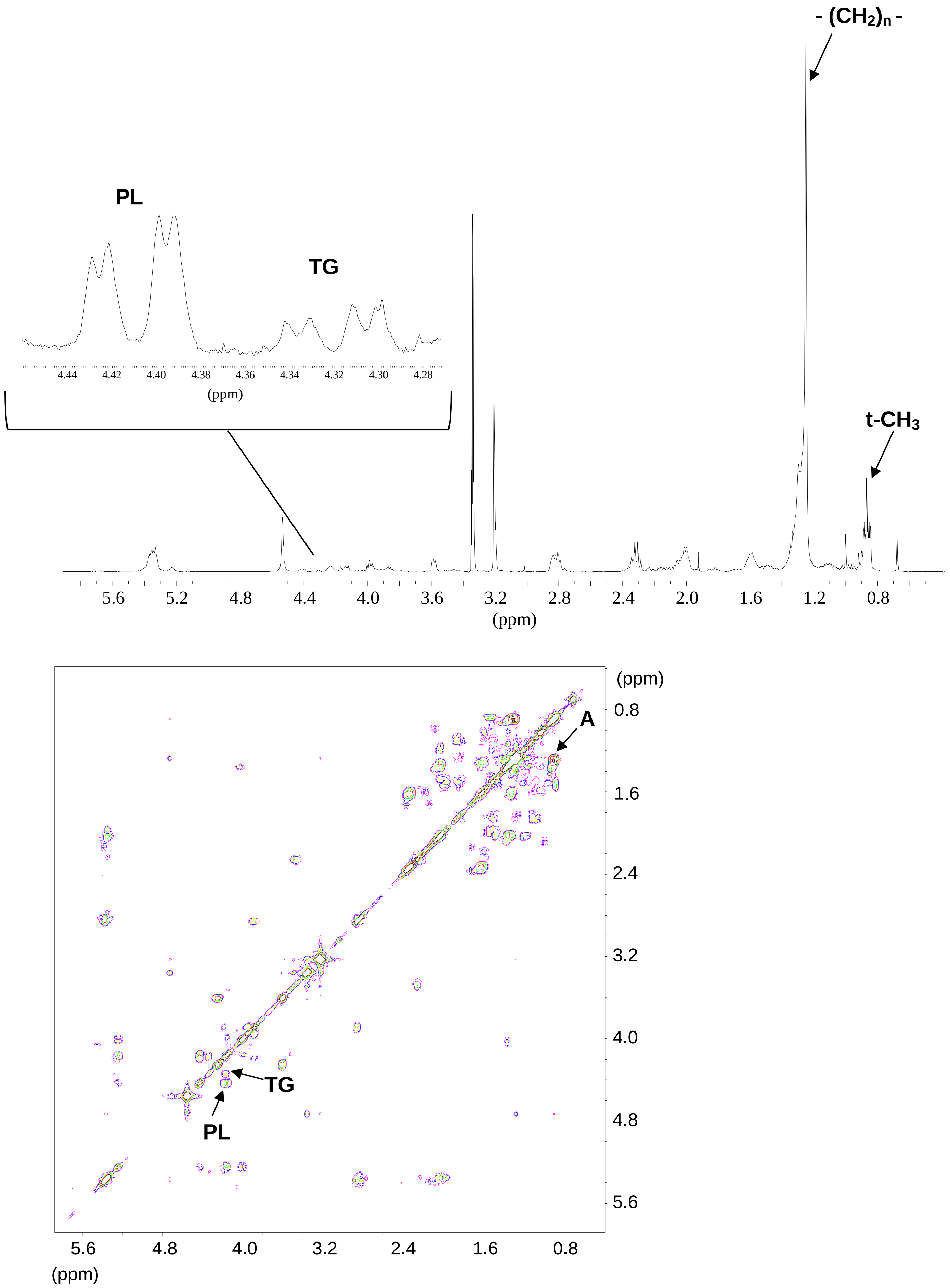

Figure 1S. 1D and 2D COSY 1H NMR spectra of extracted lipids from one representative sample of MCF-7 cell. Lipid extraction was performed as described in ref. 19. The inset in 1D spectrum represents glycerol C-2 region with glycerol proton signals of triglycerides (TG) centered at 4.32 ppm and glycerol proton signals of phosphoglycerides (PL) centered at 4.42 ppm. The corresponding cross peak of TG at 4.35-4.20 ppm and PL at 4.45-. are labelled in the 2D COSY spectrum. The cross peak A due to the interaction of terminal methyl group peak at 0.89 ppm and the proximal methylene at 1.28 ppm, was used to quantify MLs because it is representative of the corresponding bulk fatty acids chains.
