# Supplementary material for: Metabolic Study of Breast MCF-7 Tumor Spheroids after Gamma Irradiation by 1H NMR Spectroscopy and Microimaging
Source: Front Oncol. 2016 Apr 28;6:105. doi: 10.3389/fonc.2016.00105 (PMC4848320; doi:10.3389/fonc.2016.00105)
Supplement: Supplementary file 2 [file Image_2.PDF]

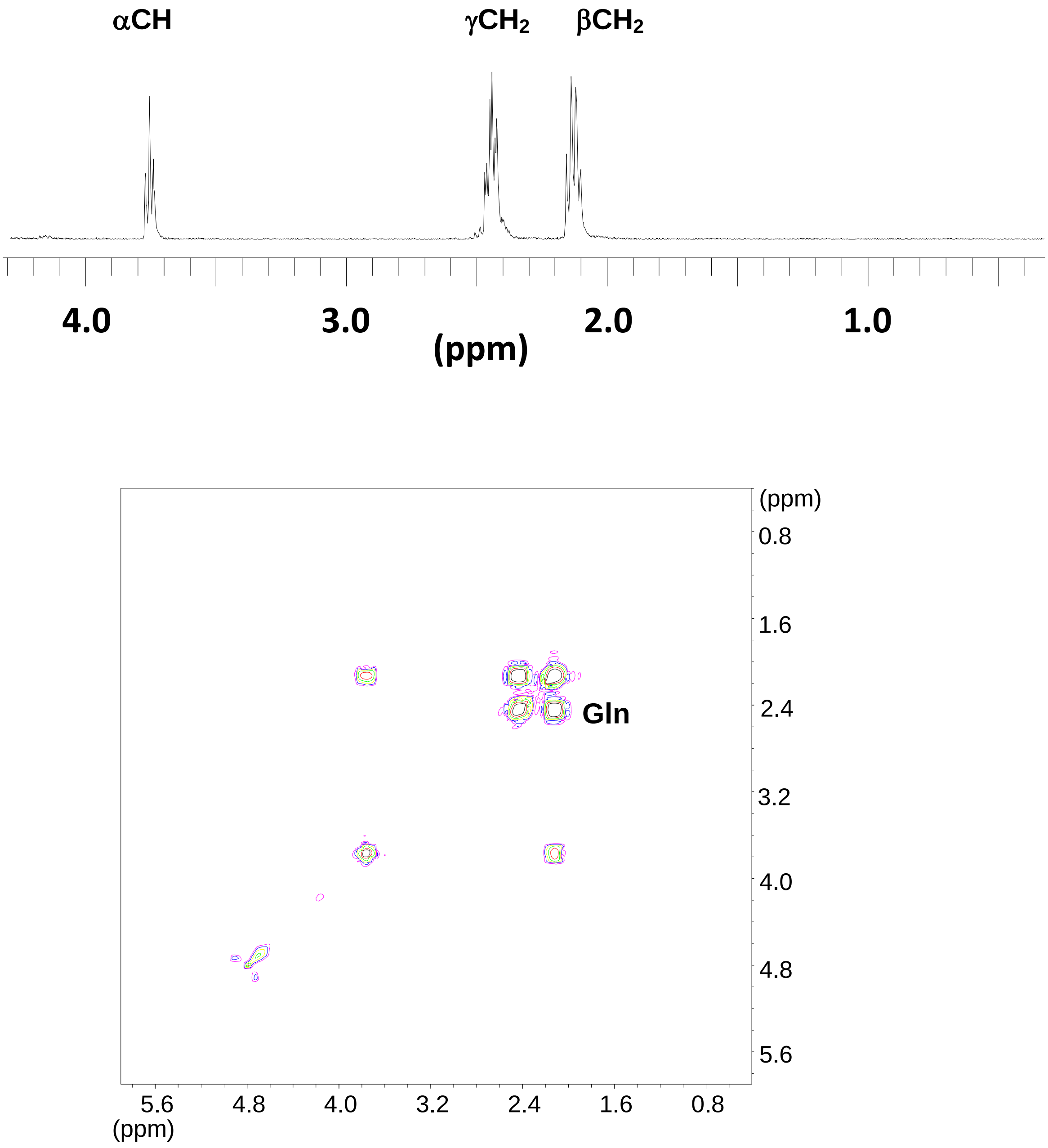

Figure 2S. 1D and 2D COSY 1H NMR spectra of glutamine (Gln) pure compound.10mM Gln pure compound was suspended in PBS. Signals of αCH at 3.78 ppm, γCH_2 at 2.46 ppm and βCH_2 at 2.14 ppm are reported. The cross peak at 2.43-2.14 ppm (label Gln), arising from the correlation between γCH_2 and βCH_2 protons, was used to quantify Gln content in cell and spheroid spectra.
